# How can we deal with stoma supplies in a resource-limited setting? The Golbasi earthquake experience

**DOI:** 10.1016/j.tcr.2025.101172

**Published:** 2025-04-14

**Authors:** Elsa Leiritz, Laurent Bernhard, Michel Orcel, Isabelle Arnaud, Philippe Agopian, Benjamin Conte, Tristan Tison, Brice Malgras, Sebastien Gaujoux

**Affiliations:** a Department of Hepatobiliary and Pancreatic Surgery and Liver Transplantation, AP-HP Pitié-Salpêtrière Hospital, Paris, France; b Service Départemental d'Incendie et de Secours (SDIS) du Haut-Rhin, Mulhouse, France; c Service Départemental d'Incendie et de Secours (SDIS) du Gard, Nîmes, France; d Department of Visceral and Digestive Surgery, Hôpital d'Instruction des Armées Bégin, Saint-Mandé, France; e Sorbonne University, Paris, France

**Keywords:** Ostomy, Supplies, Resource-limited setting, Earthquake

## Abstract

**Introduction:**

In winter 2023, two earthquakes struck southern and central Turkey, resulting in an estimated 56.000 deaths and more than 100.000 persons with injuries. The management of patients presenting post-earthquake injuries and the usual surgical emergencies was highly challenging in these extremes' conditions. This report presents a home-made temporary solution for ostomy care and supplies in a resource-limited setting.

**Case presentation:**

A 72-year-old woman, admitted 9 days after the earthquake, presented with a transverse colonic incarceration in a Morgagni-Larrey parasternal diaphragmatic hernia, associated with Hinchey III peritonitis. Extended right colectomy was performed with right ileostomy and left colostomy. A home-made ostomy was designed using as supplies an empty intravenous perfusion bag and transparent film dressings. This cheap, homemade and ready-to-use stoma supply was used during the first 3 postoperative days, without any leak or peri-ostomy skin irritation.

**Conclusion:**

In extreme perioperative condition, in a resource-limited setting, ostomy can be temporary equip using a homemade and ready-to-use stoma supply. This equipment is feasible at a low cost in all condition and fit until definitive ostomy supplies can be provided.

## Introduction

On February 6, 2023, two earthquakes (magnitudes 7.8 and 7.5) struck southern and central Turkey, resulting in an estimated 56.000 deaths and more than 100.000 persons with injuries. These earthquakes destroyed or disabled most medical facilities, seriously hampering the ability to deliver surgical care, to direct wounded but also to the unwounded population that continue to need emergency care in relation with the usual surgical emergencies. If some patients were able to be transferred to distant and unaffected hospitals, other needed immediate onsite surgery, as in the EMT-2 “ESCRIM” (*Elément de Sécurité Civile Rapide d'Intervention Médicale*), the French Civil Security's projectable field hospital, as previously reported [1]. Air-transportable, this field hospital takes part in international emergency relief missions following natural disasters. Self-sufficient at the site of its deployment, it carries out medical, surgical and obstetric activities in a 1000 m^2^ tent structure, for a period of 4 to 8 weeks. This hospital allows even under extreme conditions the surgical management of post-earthquake injuries and most common surgical emergency such as cholecystitis, appendicitis, or occlusion.

In this context, there is also a need for optimal and tailored to the local situation postoperative care, especially for patients undergoing abdominal surgery with the need of digestive stoma [2]. Indeed, if in most developed countries stoma supplies are widely available and their cost met by health systems, their availability is challenging in an early post-earthquake period or in other resource-limited settings [3,4].

This report presents a home-made temporary solution for ostomy care and supplies in a resource-limited setting.

## Case presentation

**Fig. 1:**
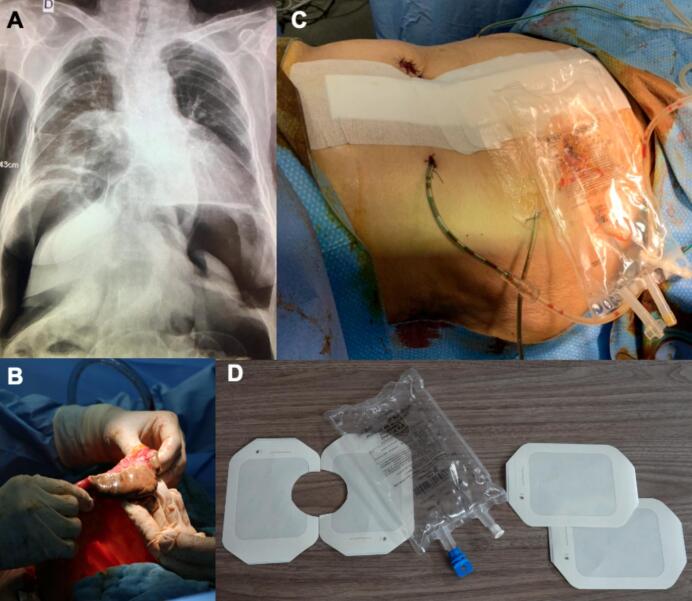
A: Preoperative X-ray showing a large pneumoperitoneum. B: Intraoperative view of the colonic necrosis. C: Immediate postoperative aspect of the stoma. D: Material needed for the stoma supplies – 1 empty 500 cc intravenous perfusion bag and 4 transparent film dressings.

Based on inpatients' medical records, we identified a 72-year-old woman, admitted the evening on the first opening day of the ESCRIM hospital, with 38.8 °C, 109 HR/min and acute abdomen. Abdominal X-ray (Fig. 1A) showed an important pneumoperitoneum associated with elevated CRP and white blood count. Median laparotomy was performed in emergency and showed a transverse colonic incarceration with necrosis (Fig. 1B) in a Morgagni-Larrey parasternal diaphragmatic hernia, associated with peritonitis. Extended right colectomy was performed with right ileostomy and left colostomy.

As shown in Fig. 1, a home-made ostomy was built using as supplies: 2 cut out 10 cm × 12 cm transparent film dressings used to protect the skin, an empty 500 cc perfusion bag, 2 additional 10 cm × 12 cm transparent film dressings used to seal and waterproof the stoma bag (Fig. 1C and D).

This cheap, homemade and ready-to-use stoma supply was used during the first postoperative days, without any leak or peri-ostomy skin irritation before her transfer to Adıyaman hospital.

## Discussion

It is well accepted that stoma confection can be a lifesaving procedure, especially during an emergency situation [2]. Nevertheless, during postoperative care and even after discharge, stoma management represents a challenge to many patients in resource-limited countries, with very limited access to stoma appliances and other accessories [4].

Only very few studies focused on stoma care and supplies in resources-limited countries, as it is resumed in Table 1. This review highlights the high rate of complications in relation with stoma in least developed countries, with mainly burnt skins troubles [14]. Occurrence of major complications was associated with patients' poor socioeconomic level and other well-known factors such as high ASA score, and ileostomy compared to colostomy [13]. One of the potential causes of this high rate of cutaneous troubles is the lack of stoma therapist and proper supplies to prevent the skin irritations [10]. Furthermore, ileostomy might require hydration and parenteral nutrition support, which are rarely available as a home-supply treatment in those countries [10]. This leads to malnutrition and wound healing disorders that in turn increase the cutaneous irritations and difficulty to correctly equip the stoma. The high rate of skin troubles and stoma leak due to the lack of correct supplies also increases the psychological and social impact of stoma, which is already known to be consequent. Patients have a modification of their self-image in addition to the important decrease of their global quality of life [9]. Long-term follow-up and education of the patients and their relatives is a way to low-down the quality-of-life impact [11,12], but is not always easy to settle up. Restoring intestinal continuity should always remain a concern after this life-saving emergency surgery. It is important to remember that, in developing country, realization of a stoma in an emergency setting remains an ethical question that should take into account social and cultural background, the possibility of restoring intestinal continuity (definitive vs temporary stoma), location of the stoma (small bowel vs colon). Therefore, in low-income countries the realization of ostoma is reserved for emergency and lifesaving procedures, most of the time in severe acute peritonitis [13]. When it is necessary to perform it, proposing a feasible and safe equipment to prevent cutaneous complications and reduce the psychological impact is mandatory.

**Table 1:** Summary of main study of ostomy supplies and care in resources-limited countries.

Country	n	Author's conclusion
Cameroon [9]	34	The realization of a digestive stoma imposes a long-term follow-up especially on the psychological level in order to allow the empowerment of the patients who all have a modification of their quality of life and their self-image.
Mali [10]	32	The assumption of responsibility of the stomies is difficult in the absence of stomatherapist, and of the high cost of the parenteral nutrition in our context.
South Asia [11]	43	The overall QOL score was considerably low in our study. The QOL was significantly associated with self-efficacy which indicates the importance of patient education and training.
Iran [12]	102	The findings demonstrated that living with stoma influences the overall aspect of QOL. Education for the patients and their families is important for improving the stoma patients' QOL.
Niger [13]	328	Acute peritonitis was the main indication of digestive ostomy. The occurrence of major complications was associated with bad socioeconomic status, ASA4 score, Altemeier class IV and ileostomy.
Tanzania [14]	167	The intestinal stomas performed at BMC are associated with various complications, which in turn, become a burden to the patients.

## Conclusion

We herein present an original cheap, homemade, and ready-to-use stoma supply that can be easily done in any basic medical or surgical facility or dispensary, to reduce the cutaneous complications and therefore the psychological and QOL impact. For stoma, various technical solutions have been proposed [[5], [6], [7], [8]]. The present case shows an alternative supply feasible in every emergency situation. Of course, the present low-cost stoma supply, still needs to be evaluated in a larger scale and other situations. It is also important to note that these solutions are for all of them temporary, and a more sustainable equipment should be found in case of long-term stoma. The issue of long-term management at affordable cost remains an issue for the largest part of the word.

## CRediT authorship contribution statement

**Elsa Leiritz:** Writing – original draft, Writing – review & editing. **Laurent Bernhard:** Visualization. **Michel Orcel:** Visualization. **Isabelle Arnaud:** Visualization. **Philippe Agopian:** Visualization. **Benjamin Conte:** Visualization. **Tristan Tison:** Visualization. **Brice Malgras:** Visualization. **Sebastien Gaujoux:** Conceptualization, Supervision, Writing – review & editing.

## Declaration of competing interest

The authors declare no conflicts of interest.
